# Impulsive-Compulsive Behaviours in Belgian-Flemish Parkinson's Disease Patients: A Questionnaire-Based Study

**DOI:** 10.1155/2019/7832487

**Published:** 2019-03-18

**Authors:** Emke Maréchal, Benjamin Denoiseux, Ellen Thys, Patrick Cras, David Crosiers

**Affiliations:** ^1^Department of Neurology, Antwerp University Hospital, Wilrijkstraat 10, 2650 Edegem, Belgium; ^2^Department of Neurology, ZNA Middelheim Hospital, Lindendreef 1, 2020 Antwerpen, Belgium; ^3^Institute Born-Bunge, Faculty of Medicine and Health Sciences, University of Antwerp, Universiteitsplein 1, 2610 Wilrijk, Belgium; ^4^Neurodegenerative Brain Diseases Group, VIB-UAntwerp Center for Molecular Neurology, Universiteitsplein 1, 2610 Wilrijk, Belgium

## Abstract

**Background:**

Impulsive-compulsive behaviours (ICB) are a potentially harmful group of behavioural symptoms among the nonmotor aspects of Parkinson's disease (PD).

**Objective:**

To develop and perform partial validation of a Belgian-Flemish version of the Questionnaire for Impulsive-Compulsive Disorders in Parkinson's Disease (QUIP) as a screening instrument for ICB in PD patients.

**Methods:**

Using a translation-backtranslation method, we developed a Belgian-Flemish version of the QUIP, which was subsequently completed by 88 PD patients. QUIP-positive patients were invited for a semistructured diagnostic interview.

**Results:**

A positive QUIP score for one or more ICB was observed in 37 patients (41%). In 15 patients (17%), a positive QUIP score for one or more impulse control disorders (ICD) was noted: pathological gambling in 1, hypersexuality in 8, compulsive shopping in 5, and compulsive eating in 8 patients. A positive QUIP score for punding, hobbyism, and/or walkabout was observed in 30 patients. The semistructured diagnostic interview was performed in 22 QUIP-positive patients. The diagnosis of ICB was confirmed in 6 patients, suggesting a positive predictive value of 27% for the Belgian-Flemish version of the QUIP.

**Conclusions:**

We have developed a Belgian-Flemish version of the QUIP, which can be used as a screening questionnaire for ICB in PD patients. Our data suggest that sensitivity is high, specificity is low, and validity of the questionnaire is similar to the original version. We confirm the necessity of additional clinical assessment of QUIP-positive patients to ascertain a diagnosis of ICB.

## 1. Introduction

Parkinson's disease (PD) is the second most common neurodegenerative brain disorder and affects approximately 1% of the population above the age of 60 years in industrialized countries [1]. Besides motor symptoms, PD patients can experience several nonmotor problems. Impulse control disorders (ICD) are a subgroup of behavioural symptoms among the nonmotor aspects of PD. These potentially devastating neuropsychiatric symptoms are characterized by failure to resist an impulse, drive, or temptation to perform an act that is harmful to the subject or to others [2]. Typical ICD in PD patients are pathological gambling (PG), hypersexuality (HS), compulsive eating (CE), and compulsive shopping (CS). In addition, compulsive behaviours, such as punding, hobbyism, and walkabout (PHW), can be observed. A particular form of behavioural disturbance is the dopamine dysregulation syndrome (DDS), in which patients develop an addictive behaviour towards dopaminergic medication. We will use the umbrella term of impulsive-compulsive behaviours (ICB) to cover the classical impulse control disorders (ICD) as well as other typical compulsive behaviours in PD patients (punding, hobbyism, and walkabout).

The prevalence of ICD in PD patients ranges from 3.5% to 42.8% [2–11]. No significant difference in prevalence was observed between drug-naïve PD patients and healthy controls [3, 12], suggesting that PD itself is not a major risk factor. The most important risk factor for developing ICD is probably the use of dopaminergic drugs, in particular dopamine agonists (DA). Higher DA dosage and longer treatment duration were associated with a higher prevalence of ICD in a longitudinal study in PD patients [13]. Additional risk factors for ICD have been reported, including male gender, younger age, levodopa therapy, personal history of smoking, preexistent history of ICD symptoms, personality profile characterized by impulsiveness and novelty-seeking, history of depression, history of substance abuse, and familial history of gambling [2, 3, 10, 14–16].

Weintraub and coworkers have validated the QUIP (Questionnaire for Impulsive-Compulsive Disorders in Parkinson's disease) as the screening instrument for ICB in PD patients [17]. We have translated the QUIP into Flemish in order to have a useful screening instrument for ICB in the Flemish-speaking patient population.

## 2. Methods

We have sent questionnaires by postal mail to 216 PD patients attending the outpatient clinic of the Antwerp University Hospital. All patients had a clinical diagnosis of Parkinson's disease according to the NINDS criteria [18] and absence of dementia was documented. The QUIP is a brief self-completed screening questionnaire. The first section assesses the following ICD: gambling, hypersexuality, and compulsive buying and eating behaviours. The second section assesses other compulsive behaviours (punding, hobbyism, and walkabout). The last section questions compulsive use of dopaminergic medication. Positive answers for each disorder are added, which leads to a maximum of five points for each of the ICD and DDS, and three points for punding, hobbyism, and walkabout.

The QUIP was translated from English to Flemish by one member of the study team. Subsequently, two different team members independently translated this first Flemish version into English. The back-translated versions were compared to the original version and any discrepancies were modified after discussion among the team members. The patients were invited to complete the QUIP and an additional questionnaire to obtain information on sociodemographic status and personal and familial medical history. Both questionnaires were completed by the patient and, if available, with help of the caregiver.

The QUIP-positive patients were invited for a more extensive interview, which was performed by one of the investigators. This semistructured interview was based on validated criteria, such as the DSM-IV criteria for gambling and binge eating [19], the Voon criteria for hypersexuality [20], and the Mc Elroy criteria for compulsive shopping [17, 21], and a standardized semistructured questionnaire was used. Three different investigators performed the diagnostic interviews, but the final interpretation was made by only 1 of those 3 investigators to reduce inter-rater bias.

We calculated levodopa equivalent daily dosage (LEDD) in milligrams according to the following conversion factors: immediate release levodopa x1; levodopa controlled release x0.75; entacapone x0.33; tolcapone x0.5; ropinirol x20; rasagiline x100; amantadine x1; levodopa-carbidopa intestinal gel x1.10; pramipexole x100; rotigotine x30; selegiline x10 [22].

Statistical analysis was performed using IBM SPSS version 20. Categorical variables were compared using Pearson's chi-squared test. Significant differences of continuous independent factors between groups were analysed using nonparametric tests. A receiver operating characteristic (ROC) curve was plotted for each ICB in the present study. We have compared the calculated area under the curve (AUC) in the present study with the analysis, which was reported for the original QUIP [17]. The sensitivity and specificity were also compared for each ICB, based on the validated cutoff values of the original questionnaire.

### 2.1. Compliance with Ethical Standards

All activities involving human participants in this study were in accordance with the ethical standards of the institutional and national research committee and with the 1964 Helsinki Declaration and its later amendments or comparable ethical standards. The study was approved by the Antwerp University Hospital Ethics Committee (reference number 11/45/343). Informed consent was obtained from all individual participants included in the study for collection of questionnaire and medical data.

## 3. Results

Completed questionnaires were obtained from more than one-third of the patients (88/216 patients), resulting in a response rate of 40.7%. The demographic and clinical features of the patients are presented in Table 1.

The mean age was 70.2 ± 9.2 (range: 44–85 years). The patients had a mean disease duration of 10.2 ± 6.8 (range: 1–43 years), and the mean age of onset was 60.2 ± 10.8 (range: 30–85 years). The majority of the patients (73/85*;* 86%) was treated with oral levodopa therapy, and slightly more than half of the patients (43/85) were treated with dopamine agonists. Mean levodopa equivalent daily dosage (LEDD) was 615 ± 353 (range: 100–1779 mg). Twenty (20/85) patients (23.5%) reported a history of mood disorder. In 13/85 patients (15.3%), a history of drug and/or alcohol abuse was noted. A familial history of mood disorder and drug and/or alcohol abuse was observed in 5/85 and in 16/85 patients, respectively.

QUIP scores, indicative for the probable presence of one or more ICB, were obtained in 37/88 patients (40.9%). In fifteen patients (17%), a positive QUIP score for one or more ICD was observed: PG in 1/88 (1.1%), HS in 8/88 (9.1%), CS in 5/88 (5.7%), and CE in 8/88 patients (1.1%). Punding, hobbyism, and/or walkabout (PHW) were present in 30/88 (34.1%) patients. We invited the QUIP-positive patients (37/88) for a more extensive interview. Fifteen (15/37) patients did not complete the confirmatory diagnostic interview and were excluded from further analysis.

The positive predictive value of the Belgian-Flemish version of the QUIP was therefore calculated in the subgroup of 73 patients, excluding the 15 patients who did not complete the diagnostic interview. In this subgroup of 73 patients, a positive QUIP score was obtained for HS in 7/73 (9.6%), for CS in 3/73 patients (4.1%), and for CE in 7/73 patients (9.6%). In 18/73 patients (24.7%), a positive QUIP score for punding was observed, and in one patient, a positive score for DDS was documented. One patient (1/73; 1.4%) who scored negative on the QUIP (and thus was not invited for the interview) was diagnosed with PG on a follow-up visit with his treating neurologist.

Among the patients who completed both the QUIP and the diagnostic interview, a correct diagnosis of ICB was confirmed in 6/22 patients. The Belgian-Flemish translation of the QUIP therefore results in a positive predictive value (PPV) of 27% for the detection of ICB and a PPV of 33.4% for the detection of ICD (Table 2). The semistructured interview was only performed in QUIP-positive patients. Therefore, we cannot make any statement concerning false-negatives, and no valid conclusions can be inferred regarding sensitivity, specificity, and negative predictive value (NPV) of the Belgian-Flemish QUIP.

## 4. Discussion

We developed a Belgian-Flemish translation of the QUIP using a translation-backtranslation method. We conducted a study in 88 PD patients using this questionnaire. In 40.9% of the patients, the presence of an ICB was suggested, whereas in 17% of the patients, a positive QUIP score for an ICD was obtained. These numbers are generally in line with the reported prevalence of ICB and ICD in previous studies, using the QUIP [2, 9, 14, 23–28].

Twenty-two QUIP-positive patients participated in a semistructured diagnostic interview. The diagnosis of ICB or ICD was confirmed in 6 of these patients. A positive predictive value of 27% was calculated for the detection of ICB by the QUIP in our study population, which is in accordance with studies using the English QUIP version. If we assume that all QUIP-negative patients would also have tested negative in the semistructured interview, we obtain a sensitivity of 100% and specificity of 76% for the Belgian-Flemish version of the QUIP. These test characteristics are in accordance with the original English questionnaire [17]. We agree that in clinical practice, QUIP-positive patients should be further assessed in order to confirm the diagnosis of ICD/ICB [12, 25].

The most prevalent ICD in our study population was hypersexuality, which was also the most frequently observed ICD in a recent review paper grouping 11 different studies [29]. The majority of QUIP false-positives were found in the punding/hobbyism/walkabout (PHW) group. According to the QUIP results, 34.1% of the patients suffered from PHW. The semistructured interview confirmed a diagnosis of punding in only 4.1% of the patients, whereas hobbyism and walkabout were confirmed in none of the patients. This observation could be partly explained by misinterpretation of the questions concerning hobbyism and walkabout. Furthermore, we should also keep in mind that the true ICD/ICB frequency in our study may remain underestimated, due to hesitation from patients and/or caregivers to acknowledge the presence of a potentially socially stigmatizing behavioural disorder. There was no systematic collection of information from caregivers or significant others, which also might contribute to this underestimation.

Our study has several other limitations, the most important one being the initial small sample size, partly due to a relatively low response rate (40.7%), as well as a low rate of patients completing the diagnostic interview (59.4%). The semistructured confirmatory diagnostic interview was only performed in QUIP-positive patients. Therefore, we were not able to perform a reliable calculation of the sensitivity, specificity, and negative predictive value for the Flemish QUIP version. We can assume that these test characteristics are likely to be comparable with the original version. We observe a rather small percentage of true ICB in our population (6/73; 8.2%) in comparison with the majority of the previously reported studies [9, 12, 14, 23–28, 30]. The observed prevalence of ICB in a population can affect the calculated PPV and NPV of the questionnaire.

Differences in prevalence reported in literature should be interpreted with care. Not all studies used a semistructured interview to verify whether QUIP-positive patients fulfilled diagnostic criteria.

Our translation of the QUIP can be used as a screening instrument for ICB/ICD in the Dutch/Flemish-speaking PD patient population. The data of this pilot study suggest that the validity of the questionnaire is similar to the original version.

## Figures and Tables

**Table 1:** Clinical characteristics of complete study population, QUIP-positive, and QUIP-negative patients.

	Total	QUIP-positive	QUIP-negative
	*N*=88	*N*=36	*N*=52
Age (years)	70.2 ± 9.2	68.3 ± 9.2	71.5 ± 9.1
M/F ratio	1.3	1.8	1.1
Onset age (years)	60.2 ± 10.8	57.4 ± 10.6	62.1 ± 10.7^*∗*^
Disease duration (years)	10.2 ± 6.8	10.9 ± 6.0	9.7 ± 7.3
Married	74/87 (85.1%)	29/36 (82.9%)	45/52 (86.5%)
Higher education	29/86 (33.7%)	10/35 (28.6%)	19/51 (37.3%)
Smoking (current or past)	26/87 (29.9%)	10/35 (28.6%)	16/52 (30.8%)
History of mood disorder	20/85 (23.5%)	10/35 (28.6%)	10/50 (20.0%)
History of substance abuse	13/85 (15.3%)	7/35 (20.0%)	6/50 (12.0%)
Familial history of mood disorder	5/85 (5.9%)	4/35 (11.4%)	1/50 (2.0%)
Familial history of substance abuse	16/85 (18.8%)	9/35 (25.7%)	7/50 (14.0%)
Levodopa-induced dyskinesia	39/82 (47.6%)	13/33 (39.4%)	26/49 (53.1%)
Levodopa	73/85 (85.9%)	30/35 (85.7%)	43/50 (86.0%)
Dopamine agonist	43/85 (50.6%)	21/35 (60.0%)	22/50 (44.0%)
LEDD (mg)	615 ± 353	671 ± 418	575 ± 298

M/F ratio: male/female ratio; QUIP: Questionnaire for Impulsive-Compulsive Disorders in Parkinson's Disease; LEDD: levodopa equivalent daily dosage. Continuous variables are shown as mean ± standard deviation; categorical or binary variables are shown as proportion and percentage; variables were compared between QUIP-positive and QUIP-negative groups. ^*∗*^*p* < 0.05.

**Table 2:** Calculation of PPV and speculative calculation of AUC, sensitivities, and specificities of the Flemish translation of QUIP in comparison to the original version validated by Weintraub and coworkers [17].

	*N*	AUC (ref. value original)	Cutoff	Sensitivity (ref. value original)	Specificity (ref. value original)	PPV (ref. value original)
All ICB (ICD + PHW + DDS)	73	0.881^*∗*^(0.85)	—	1 (0.96)	0.76 (0.73)	0.27 (0.62)
All ICD	73	0.841^*∗*^(0.88)	—	0.8 (0.97)	0.88 (0.79)	0.34 (0.53)
Gambling	73	0.493	2	0 (0.91)	0.98 (0.97)	0 (0.71)
Hypersexuality	73	0.971^*∗*^	1	1.0 (1.0)	0.94 (0.89)	0.43 (0.47)
Compulsive shopping^*∗∗∗*^	73	—	1	—(0.8)	—(0.89)	—(0.33)
Binge eating	73	0.846^*∗∗*^	2	0.75 (0.86)	0.94 (0.89)	0.43 (0.26)
Punding, hobbyism, or walkabout	73	0.893^*∗∗*^	1	1 (0.6–0.96)	0.79 (0.9–0.97)	0.17 (0.43–0.61)
DDS^*∗∗∗*^	73	—	—	—	—	—

ICB: impulsive-compulsive behaviours; ICD: impulse control disorders; PHW: punding, hobbyism, and walkabout; DDS: dopamine dysregulation syndrome; AUC: area under the curve; PPV: positive predictive value. ^*∗*^*p* < 0.01; ^*∗∗*^ *p* < 0.05; ^*∗∗∗*^no presence of this disorder was detected in our study population.

## Data Availability

The data used to support the findings of this study are restricted by the Institutional Review Board of the Antwerp University Hospital according to local regulations, in order to protect patient privacy. Data are available, by contacting the corresponding author, for researchers who meet the criteria for access to confidential data.
